# Giant spontaneous epidermal cyst of the labia minora: a rare case report and literature review

**DOI:** 10.3389/fonc.2025.1695646

**Published:** 2025-10-09

**Authors:** Xiang Li, Yuanyuan Bai, Jie Li, Ruili Li, Jinying Fu

**Affiliations:** ^1^ The Second Clinical Medical School, Henan University of Chinese Medicine, Zhengzhou, Henan, China; ^2^ Department of Gynaecology, Henan Province Hospital of Traditional Chinese Medicine, Zhengzhou, Henan, China

**Keywords:** epidermal cyst, labia minora, surgery, epidermal inclusion cyst, case report

## Abstract

**Background:**

Epidermal cysts are benign subcutaneous masses resulting from the implantation of epithelial cells, following trauma or surgery. Giant spontaneous epidermal cysts of the labia minora are rare. To date, only 3 cases of epidermal cysts located in the labia minora have been reported worldwide.

**Case description:**

This study presents a case involving an epidermal cyst of the labia minora with a diameter exceeding 8 cm, accompanied by endometriosis within an episiotomy scar and a concurrent Bartholins’s cyst. All lesions were surgically excised, successfully resolving the patient’s symptoms, and the final pathological diagnosis confirmed an epidermal cyst and endometriosis. The patient recovered well postoperatively, and follow-up at 3 months revealed no discomfort or complications.

**Conclusion:**

This case shows that spontaneous epidermal cysts of the labia minora are very rare and can enlarge rapidly over a short period. Prompt and thorough surgical excision is recommended to prevent recurrence and the potential for malignant transformation.

## 1 Introduction

Epidermal cysts (EC), also known as epidermal inclusion cysts, are intradermal or subcutaneous tumors contained within the epidermis, formed as a result of invagination of keratinized squamous epithelium. ECs are firm to fluctuant, dome-shaped masses that can be solitary or multiple lesions. They are generally less than 1 cm in diameter, grow slowly, and cease enlarging upon reaching 5 cm. Although ECs are often associated with trauma or surgical injury, spontaneous and giant cases are rare (1). These cysts frequently occur on the head, neck, back, and limbs (2). Vulvar ECs are typically located on the labia majora and clitoris. However, occurrence on the labia minora is rare, with the first case reported by Mustafa Pehlivan in 2015 (3–5).

We present the case of a giant spontaneous EC in the labia minora that led to secondary sexual dysfunction. This article reviews the clinical, imaging, and pathological characteristics, as well as the diagnosis, management, and prognosis of this condition. Previously reported cases are also summarized. This manuscript follows the CARE guidelines to ensure the accuracy and transparency of the presented information.

## 2 Case description

A 38-year-old woman was admitted for treatment of a vulvar mass that had been present for 2 years. Initially measuring about 0.3 × 0.5 cm, the lesion gradually enlarged without any treatment, resulting in discomfort during ambulation, significant emotional burden, and impaired sexual function. She complained of pain at the episiotomy scar, exacerbated during menstruation. Another painless cystic mass was palpable below the right labia majora. Her history included a mediolateral episiotomy during vaginal delivery in 2012, with no other significant surgical, trauma, personal, or family medical history.

**Figure 1:**
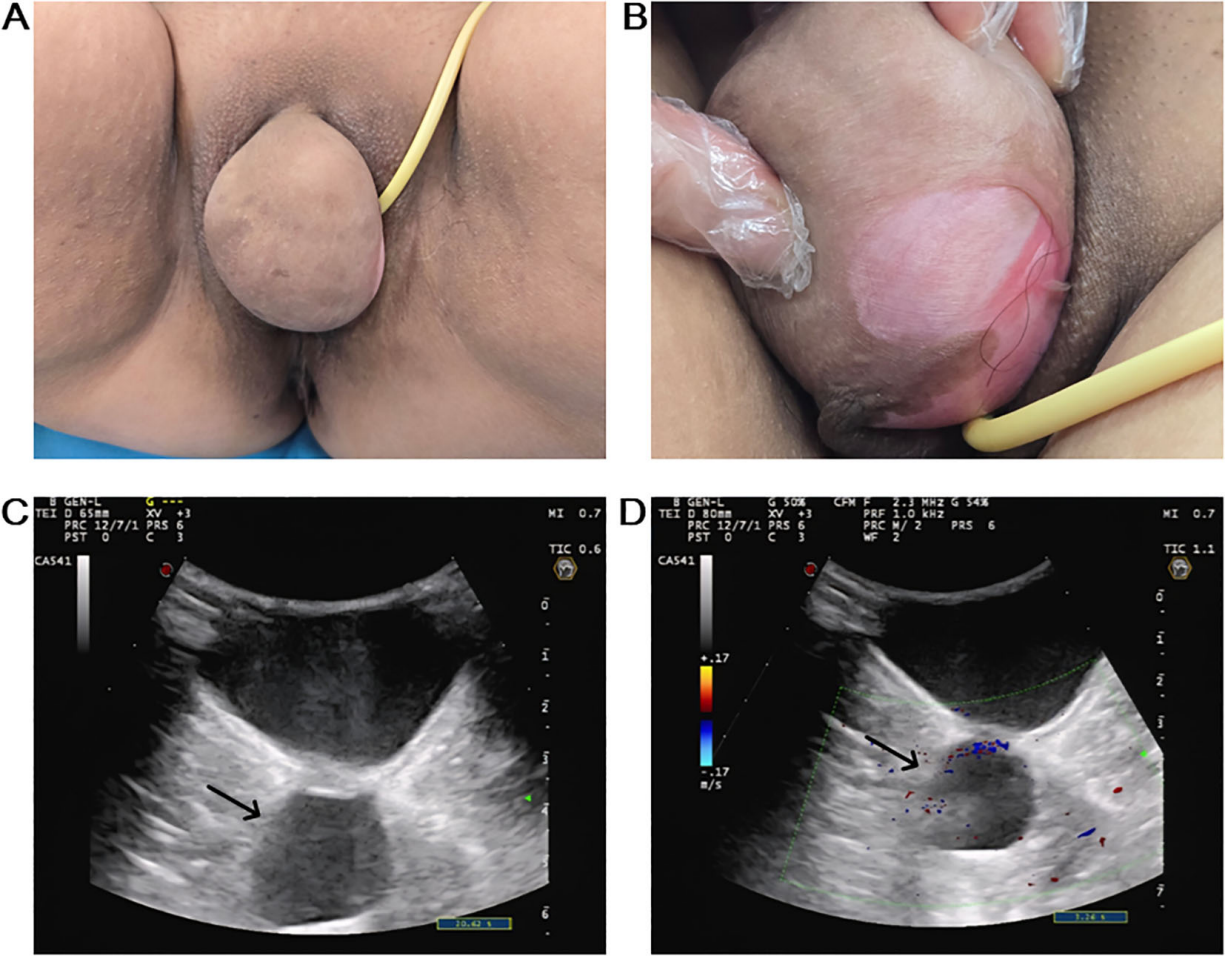
Preoperative clinical and imaging findings. **(A)** A giant cystic mass in the right upper labia minora obscured the urethral meatus and vaginal orifice. **(B)** The cyst resulted in abnormal cutaneous manifestations and lesions. **(C)** US showed a 8.2 × 5.8 × 3.9 cm, well defined, hypoechoic cystic mass within the right labia minora (black arrow). **(D)** Color Doppler flow imaging demonstrated no significant flow signal within the cyst (black arrow).

Gynecological examination revealed an approximately 8.0 × 7.0 × 5.0 cm, non-tender, well-defined cystic mass in the superior right labia minora. This mass caused deformation, overstretched and broken skin folds, and a reddish lesion (**Figures 1A, B**). Deep within the inferior right labia majora, a movable, non-tender, 4.0 × 3.5 cm cystic mass was identified. In contrast, a firm, tender, and fixed 4.0 × 4.0 cm mass was palpated at the episiotomy site.

Color Doppler ultrasonography (US) detected multiple vulvar cystic masses, consistent with multiple Bartholin’s cysts. The largest cyst measured about 8.2 × 5.8 × 3.9 cm, and another measured 3.8 × 2.6 × 2.3 cm. Both lesions exhibited well-defined margins, suboptimal internal echogenicity with dense particulate sediment, and no significant internal vascular flow (**Figures 1C, D**). Human papillomavirus (HPV) testing and ThinPrep cytologic test results were normal.

**Figure 2:**
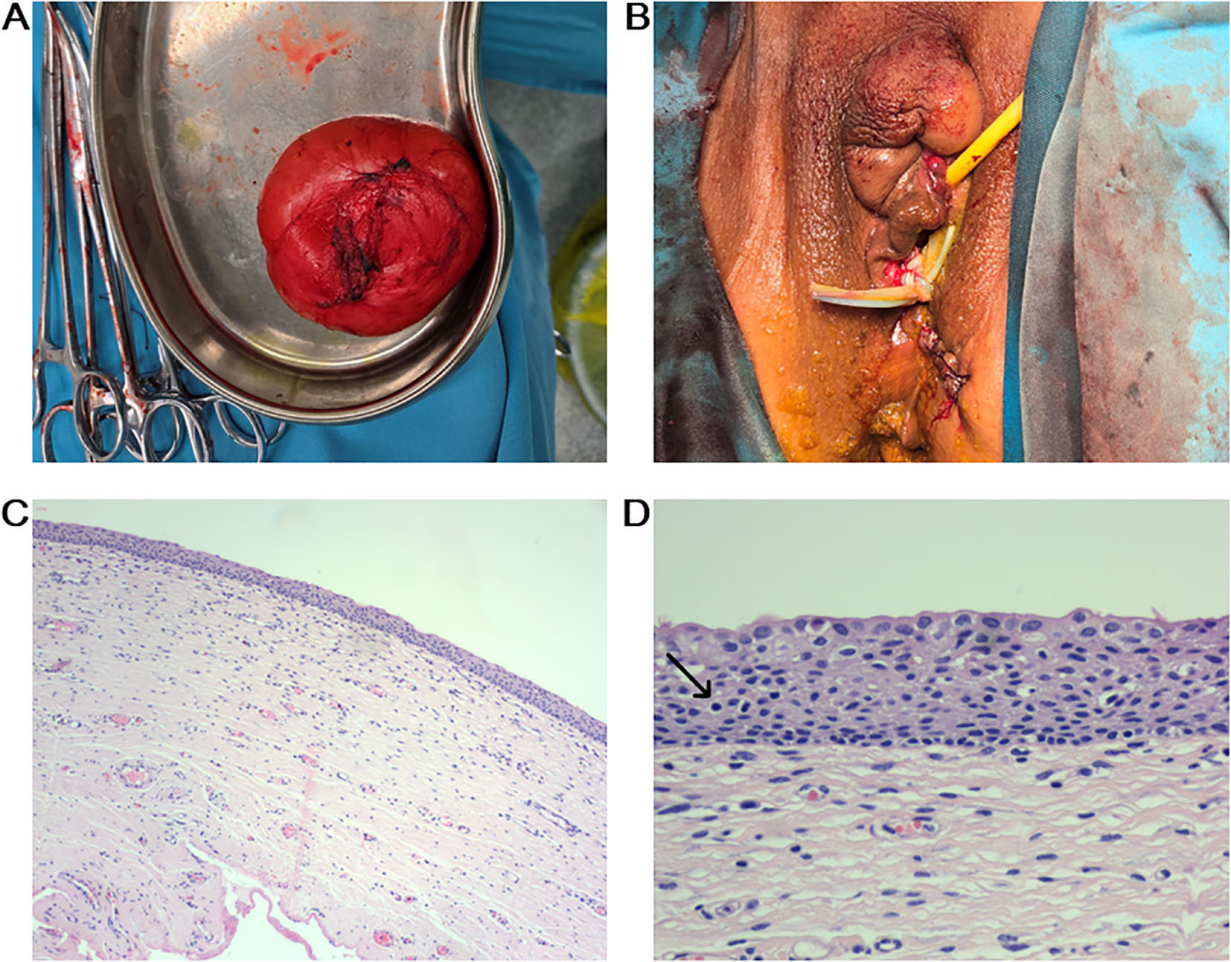
Postoperative outcome and H&E staining. **(A)** The cystic mass was completely excised. **(B)** All lesions were excised without damaging to the vagina or urethra. **(C)** Low power view (10×) showing cyst wall. **(D)** High power view (40×) revealed the cystic lesion was lined by stratified squamous epithelium, consisting of a basal layer, spinous layer, and granular layer, with scattered lymphocytic infiltration (black arrow).

After obtaining informed consent, surgical excision was performed under general anesthesia. First, an incision was made along the medial superior right labia minora, over the point of maximal cystic fluctuation. The cyst was exposed and completely enucleated using blunt and sharp dissection (**Figures 2A, B**). The cyst contents appeared yellowish, turbid, and malodorous. Hemostasis of the wound bed was achieved using electrocautery, followed by suture closure. Next, the mass in the inferior right labia majora underwent incision, irrigation, and drainage. Finally, an elliptical excision along the episiotomy scar removed a bluish-purple lesion containing old hemorrhage. Hemostasis was secured with electrocautery, and the wound was sutured.

Pathological examination revealed that the incised cystic mass had a wall thickness of 0.1 cm and contained celiac fluid. Microscopically, the cyst wall was covered with layers of squamous epithelium, including the basal, spinous, and granular layers, along with a small amount of lymphocyte infiltration (**Figures 2C, D**).

In summary, the patient was diagnosed with EC of the labia minora, endometriosis within an episiotomy scar and a Bartholin’s cyst. Postoperatively, the patient improved with treatment including antibiotics, wound care, and oral Dienogest tablets, and was subsequently discharged. By 3 months postoperatively, the incision was well-healed without recurrence or complications. Sexual dysfunction resolved and uncomfortable symptoms disappeared. Follow-up US showed no abnormal lesions at the surgical site.

## 3 Discussion

ECs, first described by Cruveilhier in 1835, predominantly affect children and young adults, with a male predominance (2:1), and commonly arise on the scalp, face, neck, trunk, or buttocks. Involvement of the reproductive system is unusual, and occurrence specifically on the labia minora is rare, with only 3 prior cases reported worldwide (**Table 1**) (5–7). Our case represents the largest and most complex spontaneous case of EC occurring on the labia minora. The current case represents the largest documented lesion to date and is unique in its coexistence with endometriosis and a Bartholin’s cyst.

**Table 1:** Summarizing of labial minora epidermal cyst.

References	No.	Age	Course	PSH	TH	Size	Location	US	CT	PD	Treatment	Prognosis
Pehlivan M, et al(2015) (5)	Case 1	47	1 yr	None	None	6 cm	Left labia minora	−	−	Vulvar epidermoid cyst	Surgical removal	Full recovery
Ruiz J(2024) (6)	Case 2	40	13 yr	None	None	4.5 cm	Right labia minora	−	−	Benign epidermal inclusion cyst	Surgical excision	Full recovery
Al-Jawad M,et al(2024) (7)	Case 3	54	−	None	None	7 cm	Right labia minora	Well-defined cystic mass with no clear vascularity	Formation of liquid density	Epidermal cyst	Surgical excision	Full recovery

PSH, Past surgical history; TH, Trauma history; PD, Pathological diagnosis.

The pathogenesis of ECs remains unclear, with several theories proposed. Firstly, the embryonic ectopic ectodermal tissue development theory posits that misplaced ectodermal cells during embryogenesis migrate into deeper tissues, persist, and proliferate to form ECs (8). Secondly, the epidermal implantation theory suggests epidermal fragments implanted into the dermis or subcutaneous tissue after trauma, infection, or surgery can proliferate and form ECs (9). Thirdly, polycystic ECs are mostly associated with genetic syndromes such as Gardner syndrome, Favre-Racouchot syndrome, and Gorlin syndrome (10). Last but not least, HPV types 6/11 have been observed in the spinous layer of the cyst wall of scrotal ECs in recent years, suggesting that HPV is a potential etiologic agent (11). In this case, the EC was located away from the previous episiotomy site, with no history of trauma, surgery, or HPV infection. Therefore, the etiology is more consistent with the first theory.

ECs typically appear on US as well-defined, hypoechoic or anechoic cysts without internal vascularity. While the US findings in this case were consistent with the above description, the lesions were misinterpreted as multiple Bartholin’s cysts, leading to the diagnosis of EC not being taken into account preoperatively. Simple Bartholin’s cysts typically appear on US as round or oval lesions with well-defined margins, hypoechoic or anechoic content and an absence of internal vascular flow, which is similar to ECs. However, due to the high fluid content of the Bartholin’s cysts, they showed increased through-transmission, a feature that can help distinguish them from ECs (12). On computed tomography (CT), ECs appear as thin-walled cystic lesions of fluid density, demonstrating a round or ovoid shape with smooth borders. They may be unilocular or multilocular, encapsulating a heterogeneous mixture of cholesterol and keratin (9). Magnetic resonance imaging (MRI) plays a crucial role in the preoperative diagnosis, differential diagnosis, and surgical decision making for ECs. MRI characteristics of ECs include well-circumscribed, round or oval lesions. Generally affected by the maturity, denseness, and keratin content of the cyst, T1WI shows low or equal signal, and T2WI shows obvious medium-high signal features. Restricted diffusion is a typical MRI feature of ECs. These signs are valuable in distinguishing them from neoplastic lesions (13). Lipomas share similar clinical presentations with ECs, typically manifesting as painless, slow-growing soft tissue masses. While lipomas classically appear as circumscribed, lobulated, lipomatous masses on MRI. They demonstrate mild T1WI hypointensity relative to subcutaneous fat, incomplete fat suppression on short TI inversion-recovery sequences, and heterogeneous enhancement. Enhancing internal septations and vascular structures may also be observed within the lesion (14).

Histopathology remains the diagnostic gold standard, revealing a cyst lined by stratified squamous epithelium without adnexal structures, filled with keratinous debris. Keratinocytes continuously desquamate and accumulate within the cyst lumen, often appearing as grayish-white or yellowish, caseous necrotic materials. When sufficiently enlarged, the cyst may rupture, releasing keratin into surrounding tissues and causing acute inflammatory granulomas. While the EC in the patient was remarkably large, approaching 10 cm in diameter, it fortunately neither ruptured nor became cancerous. Microscopic examination revealed the cyst wall lined by stratified squamous epithelium exhibiting the basal layer, spinous layer, and granular layer, along with scattered lymphocytic infiltration. Histopathological analysis allows for differentiation from conditions such as endometriosis, dermoid cysts, lipomas, pilomatricomas, and cutaneous squamous cell carcinoma with cystic degeneration (15).

Surgical excision is the preferred treatment for giant ECs and is essential for preventing recurrence and malignancy. Preoperative puncture biopsy of the cyst is contraindicated to avoid increasing the risk of infection or malignant tumor dissemination. The incision length should approximate the maximum diameter of the cyst to ensure its complete enucleation. Intraoperative rupture of the cyst wall, retention and spillage of contents into the surrounding tissue should be avoided to minimize complications. The cyst cavity should be sutured intermittently from the base without leaving an empty cavity to avoid hematoma formation by blood leakage from the venous plexus at the base of the cyst. For infected cysts that have ruptured, extensive and thorough debridement of the infected lesion is mandatory. Surgical excision of giant ECs in the labia minora presents specific challenges. Firstly, this region is rich in blood vessels and nerves, which should be protected intraoperatively. The relationship between the cyst and surrounding tissues must be clearly delineated to avoid iatrogenic injury and significant hemorrhage. Secondly, postoperative cosmesis must be considered to restore both physiological function and the natural anatomical appearance of the vulva. Appropriate trimming of redundant skin and tension-free suturing facilitates spontaneous skin contraction and wound healing, ultimately yielding optimal cosmetic outcomes (3, 4, 7). In this case, the EC was located on the superior right labia minora, adjacent to the clitoris. Enucleation was performed via combined blunt and sharp dissection along the outer layer of the cyst wall. Following complete cyst removal, the subcutaneous tissue was closed with interrupted sutures and there was little intraoperative bleeding. However, due to limited experience with ECs surgery in this location, the overstretched skin was not trimmed, resulting in a suboptimal cosmetic outcome. In addition to traditional complete excision, smaller lesions may be managed by laser, photodynamic therapy, electrosurgery, or minimal drainage, whereas multiple lesions may be excised in stages (16–18). While the majority of ECs are benign and indolent, rare malignant transformation to squamous cell carcinoma has been reported, particularly in large lesions (19). The probability of cancerous transformation of ECs with a diameter of more than 5 cm reaches 20% (20). Thus, timely excision of giant ECs is strongly recommended.

## 4 Conclusion

Giant spontaneous ECs of the labia minora are rare and often misdiagnosed preoperatively, resulting in inappropriate management. While medical imaging examinations lack sufficient sensitivity and specificity for accurate diagnosis, histopathological examination remains the gold standard. Surgical excision is the primary treatment, and early intervention is recommended to prevent cosmetic and functional complications. Clinicians should remain vigilant for atypical presentations and consider ECs in the differential diagnosis of vulvar masses.

## Data Availability

The original contributions presented in the study are included in the article/supplementary material. Further inquiries can be directed to the corresponding authors.

## References

[1] Birge O Erkan MM Serin AN. Case report: epidermoid inclusion cyst of the clitoris as a long-term complication of female genital mutilation. J Med Case Rep. (2019) 13:109. doi: 10.1186/s13256-019-2035-6, PMID: 31027516 PMC6486694

[2] Nigam JS Bharti JN Nair V Gargade CB Deshpande AH Dey B. Epidermal cysts: A clinicopathological analysis with emphasis on unusual findings. Int J Trichology. (2017) 9:108–12. doi: 10.4103/ijt.ijt_16_17, PMID: 28932061 PMC5596644

[3] Yang WC Huang WC Yang JM Lee FK. Successful management of a giant primary epidermoid cyst arising in the labia majora. Taiwan J Obstet Gynecol. (2012) 51:112–14. doi: 10.1016/j.tjog.2012.01.023, PMID: 22482981

[4] DiCarlo-Meacham AM Dengler KL Snitchler AN Gruber DD. Clitoral epidermal inclusion cyst leading to anorgasmia: A case report and literature review. J Pediatr Adolesc Gynecol. (2020) 33:321–23. doi: 10.1016/j.jpag.2020.01.150, PMID: 32028052

[5] Pehlivan M Ozbay PO Temur M Yilmaz O Gumus Z Guzel A. Epidermal cyst in an unusual site: A case report. Int J Surg Case Rep. (2015) 8C:114–16. doi: 10.1016/j.ijscr.2015.01.001, PMID: 25658206 PMC4353988

[6] Ruiz J. Large labia minora epidermal inclusion cyst. BMJ Case Rep. (2024) 17. doi: 10.1136/bcr-2023-257949, PMID: 38320827 PMC10860003

[7] Al-Jawad M Barakat M Dahoud S Lbabidi NA Kawas A Fattal F. Vulvar epidermal cyst in a 54-year-old virgin female: A very rare case report. Int J Surg Case Rep. (2024) 123:110226. doi: 10.1016/j.ijscr.2024.110226, PMID: 39213927 PMC11402154

[8] Sritharan K Ghani Y Thompson H. An unusual encounter of an epidermoid cyst. BMJ Case Rep. (2014) 2014. doi: 10.1136/bcr-2014-204186, PMID: 24825558 PMC4025405

[9] Sun PM Yang HM Zhao Y Yang JW Yan HF Liu JX. Contrast-enhanced computed tomography findings of a huge perianal epidermoid cyst: A case report. World J Clin cases. (2019) 7:3778–83. doi: 10.12998/wjcc.v7.i22.3778, PMID: 31799304 PMC6887621

[10] Koh KJ Park HN Kim KA. Gardner syndrome associated with multiple osteomas, intestinal polyposis, and epidermoid cysts. Imaging Sci Dent. (2016) 46:267–72. doi: 10.5624/isd.2016.46.4.267, PMID: 28035305 PMC5192025

[11] Kawase M Egawa K Ishiji T Nakagawa H. Human papillomavirus type 6/11 identified in an epidermoid cyst of the scrotum. J Dermatol. (2018) 45:224–27. doi: 10.1111/1346-8138.14089, PMID: 28983946

[12] Eppel W Frigo P Worda C Bettelheim D. Ultrasound imaging of Bartholin’s cysts. Gynecol Obstet Invest. (2000) 49:179–82. doi: 10.1159/000010242, PMID: 10729758

[13] Wu P Wang C Jiang Y Zhang Z Gao J Fan Z. Diagnosis and therapy of giant epidermoid double cysts with infection on the buttock: A case report and literature review. Med (Baltimore). (2024) 103:e37193. doi: 10.1097/MD.0000000000037193, PMID: 38335398 PMC10861019

[14] Zhao JM Tafti D Kao E Schwope R. A rare case of vulvar hibernoma treated with resection. Cureus. (2020) 12:e9111. doi: 10.7759/cureus.9111, PMID: 32789056 PMC7417086

[15] Lin T Ding L. Primary intracranial squamous cell carcinoma arising in epidermoid cysts: A case report and review of literature. Med (Baltimore). (2025) 104:e42094. doi: 10.1097/MD.0000000000042094, PMID: 40258769 PMC12014124

[16] Park SW Choi J Lee HS Kim J. Minimal incision suction-assisted excision of a large epidermal cyst. Aesthetic Plast Surg. (2015) 39:570–73. doi: 10.1007/s00266-015-0517-5, PMID: 26085226

[17] Zhang LC Hao LM Tan JX Huang YB Huang HF Hu J. Efficacy of the combination of minimally invasive CO(2) laser incision with photodynamic therapy for infected epidermoid cysts. Photodiagnosis Photodyn Ther. (2020) 30:101791. doi: 10.1016/j.pdpdt.2020.101791, PMID: 32344196

[18] Diep M Cassarino DS. Multiple scrotal cysts composed of combined syringomas and epidermal inclusion cysts: A previously unreported association. Am J Dermatopathol. (2020) 42:52–4. doi: 10.1097/DAD.0000000000001489, PMID: 31361615

[19] Park BS Shin DH Kim SH Jung HJ Son GM Kim HS. Perineal squamous cell carcinoma arising from an epidermal cyst: a case report. World J Surg Oncol. (2018) 16:155. doi: 10.1186/s12957-018-1442-2, PMID: 30055637 PMC6064627

[20] Morgan MB Stevens GL Somach S Tannenbaum M. Carcinoma arising in epidermoid cyst: a case series and aetiological investigation of human papillomavirus. Br J Dermatol. (2001) 145:505–06. doi: 10.1046/j.1365-2133.2001.04400.x, PMID: 11531847

